# Emerging Evidence concerning the Role of Sirtuins in Sepsis

**DOI:** 10.1155/2018/5489571

**Published:** 2018-11-08

**Authors:** Lulan Li, Zhongqing Chen, Weijun Fu, Shumin Cai, Zhenhua Zeng

**Affiliations:** ^1^Department of Critical Care Medicine, Nanfang Hospital, Southern Medical University, No. 1023, South Shatai Road, Baiyun District, Guangzhou 510515, Guangdong, China; ^2^Guangdong Provincial Key Laboratory of Shock and Microcirculation, School of Basic Medical Sciences, Southern Medical University, No. 1023, South Shatai Road, Baiyun District, Guangzhou 510515, Guangdong, China

## Abstract

Sepsis, a dysregulated host response to infection, is a major public health concern. Though experimental and clinical studies relating to sepsis are increasing, the mechanism of sepsis is not completely understood. To date, numerous studies have shown that sirtuins (silent mating type information regulation 2 homolog), which belong to the class III histone deacetylases, may have a varied, or even opposite, effect in the pathogenesis of sepsis. Notably, downstream mechanisms of sirtuins are not fully understood. The sirtuin family consists of sirtuins 1–7; among them, sirtuin 1 (SIRT1) is the most studied one, during the development of sepsis. Furthermore, other sirtuin members are also confirmed to be involved in the regulation of inflammatory or metabolic signaling following sepsis. In addition, sirtuins may interact with each other to form a precise regulatory mechanism in different phases of sepsis. Therefore, in this review, by accumulating data from PubMed, we intend to explain the role of sirtuin in sepsis, which we hope will pave the way for further experimental study and the potential future clinical applications of sirtuins.

## 1. Introduction of Sirtuin Family Members

The first gene member of the sirtuin family, silent information regulator 2 (SIR2), is highly conserved from bacteria to humans. More than 35 years ago, it was first described as the mating-type regulator 1 in *S cerevisiae*. The sirtuin family proteins have been all identified as mammalian SIR2 orthologs and are widely expressed in fetal and adult tissues. To date, the superfamily of histone deacetylases (HDACs) consists of 18 members in mammals, 11 Zn-dependent HDACs (HDAC1-11) and 7 NAD^+^-dependent sirtuins (SIRT1–7) [1]. Yet, the roles of sirtuins in physiological and pathological processes are far from being fully elucidated.

Interestingly, members of the mammalian sirtuin family have different roles depending on their cell location, enzymatic activity, and substrate specificity. SIRT1 and SIRT6 are mainly located in the nucleus, whereas SIRT2 is mainly present in the cytoplasm. Moreover, SIRT3–5 and SIRT7 are mitochondrial and nuclear proteins, respectively. Notably, the distribution of some isoforms (e.g., SIRT1, SIRT2, SIRT3, and SIRT5) is varied due to their specific activity [2]. The main feature of the sirtuin family members is their NAD (+)-dependent sirtuin activity. Like other HDACs, most sirtuins regulate the expression and activity of proteins by removing their acetyl groups from lysine 314 residues of histones and nonhistones. It is worth mentioning that SIRT4 has no obvious deacetylation activity and the deacetylation activity of SIRT6 is stronger than its deacetylase activity [3]. Both SIRT4 and SIRT6 work as ADP-ribosyl transferases [4, 5]. SIRT5 has a relatively weaker deacetylase activity but has efficient demalonylase, desuccinylase, and deglutarylase activities [6]. Sirtuins have numerous potential targets such as transcriptional regulators, enzymes, and structural proteins and are also in line with the fact that more than 6800 acetylation sites have been identified in mammalian proteins [7].

Sirtuins are associated with a variety of biological processes. Their NAD^+^-dependent deacetylase activity integrates sirtuins to cellular metabolic processes, such as gluconeogenesis, fatty acid metabolism, oxidative phosphorylation, amino acid metabolism, urea cycle, and mitochondrial biogenesis. Sirtuins are also connected to the cell cycle, rDNA transcription, DNA repair, telomere homeostasis, microtubule stability, neuronal development, and circadian functions [2, 8, 9]. Therefore, changes in the expression or activity profiles of sirtuins are associated with the development of metabolic (type 2 diabetes and obesity), cardiovascular, neurodegenerative (Alzheimer's, Parkinson's, and Huntington's diseases), oncologic, and other age-associated conditions [10–12]. Although there are many investigations that show sirtuins contribute to inflammatory and autoimmune diseases, their mechanism remains ambiguous. Indeed, sirtuins have either a protective or a deleterious role in experimental airway diseases, autoimmune encephalomyelitis, and arthritis [13–16], which is possibly due to different settings and strategies used to address the function of sirtuins (inhibitory drugs, si/shRNA, and germline- and tissue-specific knockouts). In contrast, redox transition during the development of sepsis may potentially be involved in the SIRT axis. A shift in oxidation to reduction may reactivate the SIRT axis. That is, when glycolysis-dependent hyperinflammation was initiated, nuclear SIRT1 levels decreased, and when glycolysis shifted to fatty acid oxidation, levels of SIRT1 were restored [17]. Besides, the oxidation of cysteine on SIRT6 [18] and SIRT2 [19] as well as the nitrosylation of SIRT1 [20] could decrease their expression, respectively, during sepsis. Considering inflammation exists at the center of the development of metabolic, immunologic, and infectious diseases, we believe that SIRT1 and its family members may play vital roles in the pathogenesis of sepsis.

## 2. SIRT1 and Sepsis

### 2.1. Beneficial Roles of SIRT1 in Sepsis

Numerous studies have shown that SIRT1 can reduce inflammatory response after sepsis (Table 1). Some studies have shown that SIRT1 may play an important part in the hyperglycemia-mediated inflammatory response. Jia et al. found that both mRNA and protein expression of SIRT1 were significantly downregulated in RAW264.7 cells under high glucose stimulation, whereas the levels of proinflammatory cytokines interleukin-1 beta (IL-1*β*) and tumor necrosis factor-alpha (TNF-*α*) were significantly upregulated. Interestingly, the reduced level of SIRT1 by high glucose was markedly upregulated by the SIRT1 activator SRT1720, while the level and the release of IL-1*β* and TNF-*α* were significantly decreased upon SRT1720 administration. However, as soon as the activity of SIRT1 was inhibited by Ex527, or when its expression was suppressed by RNA interference (RNAi), the upregulated levels and release of IL-1*β* and TNF-*α* by the high glucose concentration were further increased [22]. A similar phenomenon is reported in a model of obesity, which is also associated with lower SIRT1 expression. In sepsis, microvascular inflammation is more obvious and mortality is higher in obese mice compared with lean mice. However, through the activation of SIRT1 by chemical agonist resveratrol in septic obese mice (ob/ob) and human umbilical vein endothelial cells (HUVECs), increased SIRT1 activity, decreased leukocyte/platelet adhesion, and E-selectin/intercellular cell adhesion molecule 1(ICAM-1) expression, ultimately improving the mice survival rate [23].

Subsequent studies sought to explore the exact mechanism of SIRT1 in sepsis. As expected, SIRT1 was noted to play a role in various downstream targets via its deacetylation activity. Among these downstream targets, the inflammation-related nuclear factor kappa-light-chain-enhancer of activated B-cells (NF-*κ*B) signaling is of paramount importance. SIRT1 negatively regulated the activity of NF-*κ*B, thus ameliorating the NF-*κ*B mediated inflammatory response. SIRT1 blocked lipopolysaccharide- (LPS-) induced secretion of interleukin 6 (IL-6) and TNF-α in murine macrophages and protected mice from septic shock and death [21]. In a septic C57BL/6J mouse model, melatonin, a potential SIRT1 activator, blunted NF-*κ*B transcriptional activity through SIRT1-dependent NF-*κ*B deacetylation, decreased the NF-*κ*B-dependent proinflammatory response, and restored the redox balance and mitochondrial homeostasis. Thus, melatonin inhibited the NACHT, LRR, and PYD domain-containing protein 3 (NLRP3) inflammasome [25]. In addition, Yuk and his colleagues recently demonstrated that, in an LPS-stimulated macrophage model, the exact target of NF-*κ*B is p65 lysine [26]. Therefore, the negative loop of NF-*κ*B-SIRT1 may be of crucial importance for SIRT1 to exert anti-inflammatory effects.

The second deacetylation target of SIRT1 regarding its anti-inflammatory response is the high mobility group protein box 1 (HMGB1), an early alarmin in inflammatory diseases. HMGB1 is indispensable in the duration of sepsis. Importantly, hyperacetylation of HMGB1 is a necessary event leading to its secretion, a process that is also dependent on HDACs. It has been reported that SIRT1, activated by calorie restriction (CR), attenuates inflammatory response in cecal ligation and puncture (CLP) mice. Similarly, compared with normally fed mice after CLP treatment, serum cytokine and HMGB1 levels were lower in male C57BL/6N mice that underwent alternate day calorie restriction for 8 d before CLP induced sepsis. This indicated that the beneficial role of SIRT1 possibly acts to reduce HMGB1 protein expression [27]. In a CLP mouse model, SIRT1 activation inhibited the nuclear-cytoplasmic HMGB1 translocation in hepatocytes and attenuated liver injury. On the contrary, HMGB1 cytoplasmic translocation was largely aggravated by nicotinamide, a SIRT1 inhibitor. In vivo experiments showed that there was a physical interaction between SIRT1 and HMGB1 in the nucleus and that SIRT1 regulated the acetylation level of HMGB1 in response to sepsis-related liver injury. Consistent with in vivo studies, a similar phenomenon was confirmed in LPS-stimulated liver L02 cells and the beneficial effect of resveratrol was almost abrogated by SIRT1 knockdown treatment [28]. Walko et al. confirmed that accumulation of HMGB1 in the nucleus and a decrease of extracellular secretion after SIRT1 activation were found in LPS-induced human acute monocyte leukemia cell lines (THP-1), mouse bone-marrow derived macrophages, and CLP mice. The abovementioned results indicated that SIRT1-mediated HMGB1 nuclear-cytoplasmic translocation may be an important mechanism to induce septic inflammatory response. Subsequently, the exact deacetylation site of HMGB1 was explored in murine macrophage RAW264.7 cells [29]. Under normal conditions, SIRT1 and HMGB1 formed a stable complex; when SIRT1 was activated, it directly interacted with HMGB1 through its N-terminal lysine residues [24, 29, 30], therefore inhibiting HMGB1 release and improving the survival rate in septic mice. On the other hand, proinflammatory substances, such as LPS and TNF-*α*, accelerate the acetylation of HMGB1 and promote the dissociation of HMGB1 and SIRT1, as well as cause a nuclear shift of HMGB1. However, when the acetylated lysine was replaced by other amino acids (such as arginine) in vivo (K282930R), and in a hypoacetylation mutant, the survival rate of septic mice was improved, indicating that the acetylation-dependent interaction between HMGB1 and SIRT1 was critical for LPS-induced lethality [30]. Our recent work confirmed that hyperacetylation of HMGB1 occurred in a CLP mouse model after SIRT1 inactivation and that the main acetylation site was K99 and K177 (data not shown).

Other mechanisms by which SIRT1 exerts anti-inflammatory effects include inhibition of TNF-*α* transcription, repression of oxidative stress, and reduction of apoptosis. In a LPS-tolerant THP-1 cell model, the researchers showed that SIRT1 activation by resveratrol inhibited TNF-α transcription and subsequent inflammatory response [24]. Our previous study found that SIRT1 activity and protein expression were greatly reduced following sepsis, accompanied by hyperacetylated manganese superoxide dismutase (SOD2). Interestingly, SIRT1 activation by resveratrol was able to deacetylate SOD2 and restore SOD2 activity and its antioxidative function, in addition to ameliorate mitochondrial dysfunction. However, the beneficial effects of resveratrol were greatly abrogated by Ex527, a selective inhibitor of SIRT1 [31]. Moreover, after melatonin treatment in CLP mice, the deacetylation level of Forkhead box protein O1 (FoxO1) and SOD2 increased and the activity of SOD and catalase (CAT) increased while malondialdehyde (MDA) production decreased. This reduced sepsis-related brain injury [32]. SIRT1's effect on apoptosis inhibition may also depend on p53 deacetylation. When SIRT1 was activated, expression of proapoptosis protein Bax increased, which may indirectly alleviate inflammation [32]. McCall et al. reported that SIRT1 participated in and coordinated cell epigenetic changes and a bioenergetic shift in endotoxin-tolerant human monocytes and leukocytes of septic patients. In the early stages of toll-like receptor 4- (TLR4-) mediated sepsis, SIRT1 rapidly accumulated at TNF-*α* and IL-1*β* promoter regions and deacetylated the RelA/p65 lysine 310 site and nuclear histone H4 lysine 16 sites, ultimately promoting NF-*κ*B transcription termination. This sequence of events thereby prevented the body from excessive inflammation. At this preliminary stage, SIRT1 promoter binding was dependent on its cofactor, NAD (+). SIRT1 remained promoter-bound and recruited de novo-induced RelB, which resulted in endotoxin tolerance [33].

In short, SIRT1's anti-inflammatory function might be due to its multiple deacetylase targets in direct inflammation-related signaling or may otherwise be involved in an indirect mechanism.

### 2.2. Deleterious Effects of SIRT1 in Sepsis

Some scholars have suggested that SIRT1 plays different roles in the different stages of sepsis. Just as mentioned above, in a human monocyte cell model of endotoxin tolerance and human leukocytes from sepsis, SIRT1 expression was not reduced but rather increased, which may be due to the elevated NAD (+) levels. Furthermore, RelB accumulated at the TNF-α promoter region in endotoxin-tolerant sepsis blood leukocytes. However, decreased SIRT1 protein levels constantly promoted the inflammatory response [33].

In addition to the inflammatory response in the development of sepsis, metabolic shifts have recently received more attention. The early initiation phase of acute inflammation is anabolic and primarily requires glycolysis with reduced mitochondrial glucose oxidation for energy, whereas the later adaptation phase is catabolic and primarily requires fatty acid oxidation for energy. Interestingly, SIRT1 may play a critical role in metabolic shifts. In TLR4-stimulated THP-1 promonocytes, SIRT1 accelerated the shift between glycolysis and fatty acid oxidation in the early stage of the inflammatory response, thus promoting the body into the late inflammation. A similar phenomenon occurred in septic human leukocytes and splenic murine spleen cells [34]. However, the exact mechanisms must be further explored. This study indicated that SIRT1, in a later adaptive state of sepsis associated with modified bioenergetics, might contribute to immunosuppression. Subsequent research confirmed that SIRT1 inhibition could strikingly reverse post-acute phase hypoinflammation during the later stage of sepsis [35]. All mice survived after treatment with Ex527 (the selective SIRT1 inhibitor), while only 40% of mice in the nonadministered (Ex527-free) group survived. Simultaneously, due to the fact that SIRT1 inhibitors restored levels of E-selectin, ICAM-1, and P-selectin glycoprotein ligand-1(PSGL-1) on the neutrophil surface in endothelial cells, Ex527 treatment also enhanced the ability of leukocytes to adhere to the small intestine microvascular endothelium and increased intraperitoneal leukocyte accumulation. This thereby enhanced the ability of the peritoneum to clear bacteria and subsequently improved endotoxin tolerance. Other benefits of Ex527 include stabilizing blood pressure; improving microvascular blood flow; and promoting the migration of premacrophages to the spleen and bone marrow [35].

In contrast to the abovementioned opinion that SIRT1 activation plays a protective role in the initial stage but a harmful role in the later immunosuppression stage of sepsis, Fernandes et al. reported that SIRT1 inhibition, not activation, showed a constantly beneficial role in inflammatory conditions and even in the initial stage of sepsis. They found that inhibition of SIRT1 expression in LPS-induced J774 macrophages reduced the expression of related cytokines and attenuated the activity of NF-*κ*B [36]. In the early stages of sepsis (1 h after CLP), SIRT1 expression is suppressed and cytokine levels are reduced; therefore, sepsis-associated coagulation disorders are reduced, and the incidence of marrow atrophy is also reduced [37]. However, this study did not conduct an in-depth investigation of the specific mechanism of SIRT1 inhibition; therefore, this view remains to be explored. We suggest that the administration time following sepsis should not be neglected, and we will discuss it in Section 2.3 of this review. Herein, we summarize the published papers concerning the beneficial effects of SIRT1 inhibition (not activation) on sepsis treatment (Table 2), which may help us better understand the detailed mechanism and potential application of SIRT1 in the treatment of sepsis.

### 2.3. SIRT1 in Sepsis: A Double-Edged Sword

**Figure 1:**
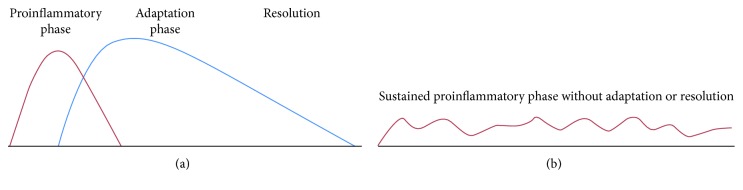
Inflammatory processes. (a) Acute systemic inflammation switches from the proinflammatory phase to the adaptive phase and eventually progresses to resolution. (b) Chronic inflammation sustains a proinflammatory phase [42].

To explain why SIRT1 almost plays an opposite role in sepsis development, we first need to review the distinct stages of this disease. Typically, acute systemic inflammation, which includes sepsis, has a distinct order and constancy [38, 39]. Its stereotypic features can be divided into phases that result in distinct clinical phenotypes: an initiation (proinflammatory) phase, an adaptive (anti-inflammatory and reparative) phase, and a resolution (restoration of homeostasis) phase [40–42]. These phases are reflected by changes from hyperinflammation to hypoinflammation to resolution in inflammatory diseases, including sepsis (Figure 1) [42].

Septic patients almost have excessive hyperinflammation during the initial stage of the disease (hours to days after the onset of sepsis), which is characterized by a cytokine storm, overproduction of reactive oxygen species (ROS), and metabolic shifts; these are crucial for disease progression [42]. To date, in this stage, SIRT1 activation has exhibited beneficial effects via three pathways: (1) deacetylation of inflammatory-related cytokines such as NF-*κ*B [25] and HMGB1 [30], leading to an attenuated inflammatory response; (2) deacetylation of oxidative stress-related factors such as FOXO1*α* [32] and SOD2 [31], thereby improving antioxidant capacity; (3) deacetylation of proapoptotic factor p53 [32], causing reduced cell apoptosis. Among these pathways, the anti-inflammatory effect should be the major pathway. However, due to the large individual differences in the time window of the inflammatory response after sepsis, administration of the SIRT1 activator, SRT1720, should insure that SIRT1 exerts a beneficial effect in a timely manner. After the initial stage, the development of the disease varies greatly from one individual to another. Some patients with sepsis (the adaptive phase is short) receive timely and effective treatment (resolution phase), while others shift from a hyperinflammatory phase to an anti-inflammatory adaptation phase, which can persist from days to weeks and is characterized by immunosuppression [43].

Excessive inflammatory reactions with multiple organ failures, followed by prolonged immunosuppression, are typical of sepsis, the most common manifestation of severe systemic inflammatory response syndrome [39]. In the immunosuppressive phase, the increased NAD (+) promotes SIRT1 expression. Elevated SIRT1 mediates deacetylation of NF-*κ*B and HMGB1, causing persistent hypoinflammation and immunosuppression. The experimental results indicated that SIRT1 expression should be inhibited in the later stage of sepsis. Due to the large individual differences in the duration of the immunosuppressive phase, the administration time for SIRT1 inhibitors is not easy to evaluate. Alterations in innate and adaptive immune cell functions, such as assessments of CD4+ T cells and T- cell subpopulations, might serve as useful indexes in the evaluation of immunosuppression [44]. To a certain extent, SIRT1 itself should be a potential index to identify the immunosuppression phase of sepsis. When the decreased SIRT1 expression begins to increase, immunosuppression occurs. In fact, in addition to SIRT1, other molecular-mediated anti-inflammatory responses may share a common contradictory application in sepsis, as a great number of inflammatory mediators exist and accompany all stages of sepsis. Therefore, we highlight the importance of monitoring the immune function of septic patients. SIRT1 should not always be activated or repressed during sepsis treatment. In addition, single-agent immune-modulatory interventions, as attempted in past sepsis trials, will likely fail. The notion of more thorough and rigorous patient selection, coupled with frequent monitoring of immune function and goal-directed immune-modulatory therapy involving multiple agents, should provide optimal clinical benefits over time [45]. Hence, the role of SIRT1 in the pathogenesis of sepsis cannot be simply defined as beneficial or detrimental but should be viewed dynamically.

## 3. Other Sirtuin Family Members in Sepsis

Other members of the sirtuin family are also involved in the development of sepsis. SIRT2 knockin mice showed decreased E-selectin and ICAM-1 levels, following attenuation of cellular adhesion with sepsis in the small intestine, as well as an improved 7-day survival rate. This study suggested that SIRT2 plays an important role in the regulation of microvascular inflammation in early sepsis [46]. However, another study found that, in fatal septic shock mice models, AGK2 (a selective SIRT2 inhibitor) administration significantly improved the survival rate of mice and decreased sepsis-related “cytokine storm” coagulopathy disorders and bone marrow atrophy [47]. In ob/ob-septic mice, SIRT2 levels were elevated during the hypoinflammatory phase (immunosuppressive phase). In in vivo experiments, SIRT2 inhibition (but not activation) led to activation of endothelial cells and circulating leukocyte, which thus reversed the inhibition of the microvascular inflammatory response. This subsequently significantly improved the survival rate. Its mechanism was related to NF-*κ*B p65 deacetylation. These results indicated that SIRT2 plays a role in the regulation of microvascular inflammatory responses in septic mice [48].

Unlike SIRT1 and SIRT2, SIRT3 activation and overexpression show several positive effects in sepsis treatment. In a CLP-induced sepsis-related acute kidney injury (AKI) mouse model, SIRT3 expression was decreased in renal tubular cells. Moreover, in a knockout mouse model, SIRT3 deletion exacerbated CLP-induced kidney dysfunction, renal tubular cell injury and apoptosis, mitochondrial dysfunction, and reactive oxygen species (ROS) production. SIRT3 deletion increased NLRP3 inflammasomes and expression of the apoptosis-associated speck-like protein, resulting in the activation of oxidative stress, and increased the production of the proinflammatory cytokines IL-1*β* and IL-18. Furthermore, it enhanced apoptosis, suggesting that SIRT3 plays a protective role against mitochondrial damage in the kidney [49]. Likewise, in CLP-induced lung dysfunction, SIRT3 expression was decreased, and intriguingly, SIRT3 knockout exacerbated vascular leakage. Conversely, SIRT3 overexpression prevented pericyte loss and vascular leakage. This study demonstrated that SIRT3 can act on perivascular cells in septic lung injury to maintain vascular integrity [50]. In sepsis-associated encephalopathy (SAE), acetylated cyclophilin D protein (CypD) expression is increased in the hippocampus of the brain. However, overexpression of SIRT3 can deacetylate CypD, thereby maintaining the integrity of the mitochondrial membrane and restoring the expression of IL-6, TNF-*α*, and caspase 3. This ultimately reduces neuroapoptosis and cognitive impairment [51]. Since the therapeutic effect of SIRT3 in sepsis is unrelated to inflammation, its activation can alleviate sepsis at any stage.

SIRT4, unlike other sirtuins, mostly functions as a histone ADP-ribosyltransferase but does not have NAD-dependent deacetylase activity [52]. In an LPS-induced endothelial dysfunction model, the expression of SIRT4 was significantly decreased, followed by elevated proinflammatory cytokines such as IL-1*β*, IL-6, and IL-8 as well as the COX-prostaglandin system (COX-2), ECM remodeling enzymes MMP-9, and the adhesion molecule ICAM-1; the increased proinflammatory cytokines aggravate inflammatory vascular diseases [53]. Furthermore, a recent study has shown that mitochondrial SIRT4 resolves immune tolerance in human monocytes by coordinately reprogramming metabolism and bioenergetics. In the human monocyte cell model of immune tolerance, both mRNA and protein expression of SIRT4 increased substantially. Conversely, enhanced SIRT4 represses fatty acid oxidation [54]. However, studies of SIRT5, SIRT6, and SIRT7 relating to the pathogenesis of sepsis are rare and need to be explored in the future.

In addition to the individual roles played by members of the sirtuin family, increased evidence has confirmed that the sirtuin family members may connect with each other and cooperate to precisely control sepsis progression. It has been reported that SIRT6 [34] and SIRT3 [55] work closely with SIRT1 in the sepsis adaptation stage to jointly regulate energy shifts, promote mitochondrial-nuclear exchange, and accelerate the development of immunosuppression. This implies that SIRT1-mediated epigenetic coordination might be a critical target in the later stages of sepsis. However, SIRT5 showed opposite expression patterns and functions compared with SIRT1 and SIRT2 in a sepsis model involving macrophages. SIRT5 deficiency decreased TLR-triggered inflammation in both the acute and the immunosuppression phases of sepsis. Interestingly, cytoplasmic SIRT5 synergized with SIRT2 and enhanced innate inflammatory responses in macrophages and even in endotoxin-tolerant macrophages by promoting p65 acetylation and activation of the NF-*κ*B pathway. Specifically, SIRT5 competed with SIRT2 to bind to NF-*κ*B p65 in a deacetylation manner. This resulted in prevention of SIRT2 binding to p65, which consequently led to increased p65 acetylation and the activation of the NF-*κ*B pathway and its downstream cytokines. Taken together, sirtuin family members in different sepsis phases can interact with each other to precisely control sepsis progression [56]. In addition, nuclear SIRT1 can guide RELB to differentially induce SIRT3 expression, and it also can increase mitochondrial biogenesis, which alters bioenergetics during sepsis adaptation. The SIRT1-RELB-SIRT3 pathway was found to be closely associated with mitochondrial biosynthesis in both TLR4-stimulated normal and septic human blood monocytes and mouse splenocytes [55].

In conclusion, sirtuin-mediated deacetylation, or other posttranslational modifications, may widely exist throughout the progression of sepsis. The interrelationship among sirtuin members widely exists and remains to be further explored.

## 4. Summary

Taken together, the NAD (+)-dependent sirtuin family is involved in the pathophysiological changes of sepsis. SIRT1/2-mediated suppression of inflammation helps to resist the disease in the initial phase and plays a diametrically opposite role in the later phase (immunosuppression phase), which indicates the importance of immune regulation in the treatment of sepsis. SIRT3 appears to be a potential target for sepsis because of its mitochondrial protective effects. Besides, by regulating inflammation and metabolism shifts, SIRT4 may also participate in the development of sepsis. However, whether SIRT5–7 is related to sepsis and how they contribute to it remains to be further researched. The sirtuin family relies mainly on its deacetylation activity and inflammation-related signals such as NF-*κ*B and HMGB1, which are the major targets of SIRT1. In addition to SIRT1, other members also play different roles during sepsis, plus their respective targets are varied. It is worth mentioning that the sirtuin family members are not independent of each other and their connections between each other can more accurately regulate the development of sepsis. This hence should be an important focus of future research.

## Figures and Tables

**Table 1:** Relationship between SIRT1 activation and sepsis-induced cell damage.

Sepsis model	Main treatment	Effects	Reference
LPS-induced macrophages or septic-shock mice	SIRT1 activation	IL-6 and TNF-α secretion is inhibited.	[21]
High glucose and LPS-induced RAW264.7 cells	SIRT1 activation	IL-1*β* and TNF-α secretion is reduced.	[22]
Septic obese mice and LPS-simulated HUVECs	SIRT1 activation	Leukocyte/platelet adhesion and E-selectin/ICAM-1 expression levels are decreased, and animal survival is improved.	[23]
LPS-induced THP1 cells	SIRT1 activation by resveratrol	Repressed transcription of TNF-*α.*	[24]
Septic mice	SIRT1 activation	NF-*κ*B is deacetylated, redox balance and mitochondrial homeostasis are restored, and NLRP3 inflammasomes are inhibited.	[25]
RAW264.7 cells	SIRT1 activation by calorie restriction	NF-*κ*B in its p65 lysine site is deacetylated.	[26]
Septic mice	SIRT1 activation	HMGB1 protein expression is reduced.	[27]
Hepatocytes from CLP mouse model/LPS-stimulated L02 cells	SIRT1 activation	HMGB1 translocation is inhibited.	[28]
THP-1 cells, murine bone marrow-derived macrophages, and CLP mice	SIRT1 activation by poly (ADP-ribose) polymerase	Increased HMGB1 nuclear retention and decreased extracellular secretion.	[29]
RAW264.7 cells and CLP mice	SIRT1 activation	Directly interacts with HMGB1 via its N-terminal lysine residues [24, 29, 30]. Inhibits HMGB1 release and improves animal survival.	[30]
Renal epithelial cells in CLP rats	SIRT1 activation by resveratrol or SRT1720	Deacetylates SOD2, reduces oxidative stress, promotes mitochondrial function, and improves animal survival.	[31]
CLP mice and septic encephalopathy	SIRT1 activation by melatonin	Deacetylates p53, FOXO1, and NF-*κ*B. Improves the survival rate, attenuates brain edema and neuronal apoptosis, and preserves BBB integrity.	[32]
Human monocyte cell model of endotoxin tolerance and human leukocytes from sepsis	SIRT1 activation	Deacetylated RelA/p65 lysine 310 and nucleosomal histone H4 lysine 16 to promote termination of NF-*κ*B dependent transcription.	[33]

**Table 2:** Relationship between SIRT1 inhibition and sepsis-induced cell damage.

Sepsis model	Main treatment	Effects	Reference
Human monocyte cell model of endotoxin tolerance and human leukocytes from sepsis	SIRT1 inhibition	Increased TNF-*α* expression and promotes the inflammatory response	[33]
TLR4-stimulated THP-1 promonocytes, blood leukocytes from septic human, and plenocytes from septic mice	SIRT1 inhibition	Decreased fatty acid oxidation	[34]
Septic mice	SIRT1 inhibition	Restored repressed endothelial E-selectin and ICAM-1 expression and PSGL-1 expression on neutrophils	[35]
LPS-stimulated J774 macrophages	SIRT1 inhibition	Reduced cytokine production and decreased NF-*κ*B activity	[36]
CLP rats	SIRT1 inhibition	Attenuates cytokine levels, induces coagulopathy, and decreases bone marrow atrophy	[37]
